# Clinical outcomes of self-expandable metallic stents for malignant obstructive atelectasis

**DOI:** 10.1038/s41598-020-60566-6

**Published:** 2020-02-27

**Authors:** Yonghua Bi, Xiaoyan Zhu, Zepeng Yu, Mengfei Yi, Xinwei Han, Jianzhuang Ren

**Affiliations:** 1 Department of Interventional Radiology, The First Affiliated Hospital of Zhengzhou University, Zhengzhou, China; 2 Department of Histology and Embryology, College of Basic Medicine, Zhengzhou University, Zhengzhou, China

**Keywords:** Chronic obstructive pulmonary disease, Surgery

## Abstract

Self-expandable metallic stents (SEMSs) have been widely used in the treatment of malignant central airway obstruction. However, few reports focus on the treatment of atelectasis and how to estimate the prior probability of success via SEMSs placement, This current study aimed to study the safety and effectiveness of SEMSs for the treatment of obstructive atelectasis, and the value of preoperative CT enhancement for ventilation of atelectasis via SEMSs placement. A total of 35 patients with obstructive atelectasis (29 male and 6 female) was included from February 2012 to March 2018. The procedures were performed under fluoroscopic guidance, and bronchoscopic laser resection was performed for severe restenosis cases after SEMSs placement. Clinical and functional pulmonary data were recorded before and 3 months after the procedure. Follow-up involved clinical data and radiographic techniques at 48 h and at 1-, 3-, 6-, and 12-month intervals. Thirty-eight SEMSs were successfully implanted in 34 patients, included 29 Y type tracheal stents, 4 small y stents, and 5 straight airway stents. After stenting, 26 cases showed full ventilation, and 3 cases were partially ventilated. The technical success and clinical success was 97.1% and 82.9%, respectively. A higher maximum enhancement CT value was found in patients with full ventilation. Mean follow-up time was 18.8 ± 4.0 months. Eight cases showed restenosis and received endoscopic laser resection, included 1 case underwent removal and 3 cases received second stenting. There were 2 cases of perioperative non-operative death, and 11 cases of post-discharge death (2 cardiac deaths and 9 malignant tumors). The survival rates of 3 months, 1 year and 2 years were 78.6%, 58.5% and 58.5%, respectively. In conclusion, SEMSs placement is safe and effective for obstructive atelectasis, and the preoperative CT enhancement played an important role in estimating the prior probability of success in the treatment of atelectasis via SEMSs placement.

## Introduction

Obstructive atelectasis caused by malignant lung or esophageal cancer is a clinical emergency, which is characterized by progressive dyspnea and risk of asphyxiation. Patients often show chronic dyspnea and difficulty to receive surgical resection, chemotherapy, and radiotherapy, thereby resulting in low quality of life and poor prognosis^1^. Although SEMSs has been widely used for palliation of airway stenosis, especially malignant central airway obstruction^2–8^. At present, few reports have described the treatment of atelectasis via SEMSs placement, except for some case reports^1,9–11^. SEMSs implantation can relieve symptoms, improve pulmonary function and stabilize cardiovascular conditions to undertake a successful surgical correction^11^. However, stent treatment also brings potential fatal complications, such as stent restenosis caused by tumor or granulation tissue, stent fracture, and stent migration^12–14^. Hachiya *et al*. even reported the potential risk of creating obstructive atelectasis in two cases after stenting^2^. Thus it is important to estimate the prior probability of success in the treatment of atelectasis via SEMSs placement. In the present study, we report our experience in the management of obstructive atelectasis using self-expandable uncovered metallic stents over a 6-year period.

## Materials and Methods

### Patient selection and study population

This retrospective study was approved by Zhengzhou university committee on human investigation. Written informed consent was obtained from all patients. All methods were performed in accordance with the relevant guidelines and regulations. The indications for SEMSs in the management of malignant obstructive atelectasis included patients who refuse or cannot tolerate surgery due to the older age, abnormal coagulation function, severe visceral dysfunction and other conditions; and patients who showed atelectasis after surgical resection for esophageal cancer and lung cancer. From February 2012 to March 2018, there were 35 patients with airway occlusion or severe stenosis complicated with obstructive atelectasis, 29 males and 6 females, with a mean age of 58.8 ± 2.1 years. All the patients underwent CT examination after admission and were definitely diagnosed as atelectasis, included 23 cases of lung cancer and 6 cases of esophageal carcinoma, and 6 cases of mediastinal carcinoma compression. Fourteen and 12 cases showed severe stenosis or occlusion in right and left main bronchi, and 9 cases in lower trachea and carina region (Table 1). All patients had different degree of dyspnea. According to Baseline Dyspnea Index (grade 0 to grade 4), there were 5 cases of grade 2, 19 cases of grade 3 and 11 cases of grade 4.

**Table 1:** The patients’ characteristics.

Characteristics	Data
Patients, No.	35
Mean age (range), years	58.8 ± 2.1 (14–79)
Male/female gender, No.	29/6
Primary disease	
Esophagus cancer	6 (17.1%)
Lung cancer	23 (65.7%)
Mediastinal carcinoma	6 (17.1%)
Airway occlusion or severe stenosis, No (%).	
Inferior trachea and trachea carina	9 (25.7%)
Right main bronchus	14 (40.0%)
Left main bronchus	12 (34.3%)
Atelectasis, No (%).	
Unilateral atelectasis (Right, Left)	7 (20.0%), 10 (28.6%)
Pulmonary lobe atelectasis	18 (51.4%)
Right upper lung	4 (11.4%)
Right middle lung	6 (17.1%)
Lower lung (Right, Left)	2 (5.7%), 6 (17.1%)
Median interval between symptom and stenting, days	30.5 (IQR:10.8,63.5)

### Airway stent types and design

Metallic stents were made of nickel-titanium alloy memory metal stents (Nanjing Micro-Tech Medical Company, Nanjing, China). SEMSs were woven with a temperature-memory nickel–titanium alloy wire, and were specially developed according to individual airway shape and size by CT measurement. All patients showed airway stenosis without fistula, and all stents were bare stents in this study. The airway stent’s diameter was 10–20% larger than the inner diameter of the trachea and main bronchus. Straight airway stents are used in patients with middle and lower trachea stenosis. Small Y stents are suitable for patients with stenosis at the proximal end of the main bronchus (Table 2).

**Table 2:** The stent types and dimensions.

Stent types	n	Median diameter (mm)	Median length (mm)
Straight airway stents	5	20 (20–22)	50 (50–60)
		MB 20 (20–28)	MB 47.5 (30–60)
Large Y type airway stents	29	LMB 12 (10–18)	LMB 47.5 (30–60)
		RMB 12 (10–18)	RMB 22.5 (10–40)
Small y type airway stents	4	MB 13 (12–18)	MB 27.5 (20–35)
		BL 10 (10-10)	BL 15 (10–40)

MB, main body; BL, bronchial limbs; LMB, left main bronchus; RMB, right main bronchus; PL, plugged limbs.

### Preoperative preparation

**Figure 1:**
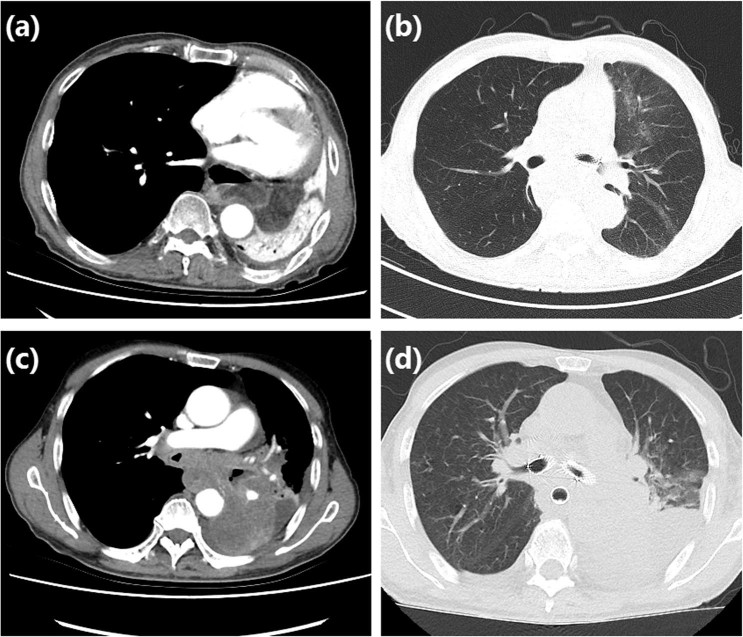
Chest CT examination. (**a**) The lung tissue of atelectasis was obviously enhanced before operation, and completely ventilated after stent placement (**b**). (**c**) The enhancement of left inferior lung tissue was poor before operation, (**d**) and the lung was not ventilated after stent.

On admission, CT scan and enhancement were performed as soon as possible to determine the location and extent of stenosis, and to assess the enhancement of atelectasis (Fig. 1). CT value increase more than 20 Hu can be defined as significant enhancement. Further tracheography was performed if necessary, such as for better showing areas of airway stenosis or occlusion for more accurate stent positioning and insertion. In addition, tracheography can also confirm the location of the catheter to avoid complications such as perforation while introduce the stiff guider wire. Half an hour before the procedure, an intramuscular injection of diazepam (10 mg) and anisodamine (Code name: 654–2, 10 mg) was administered to reduce airway secretions and calm the patient. Patients could receive preoperative injection of 10 mg of dexamethasone, to relieve dyspnea and to improve the safety and tolerability of the procedure.

### Stenting procedure

**Figure 2:**
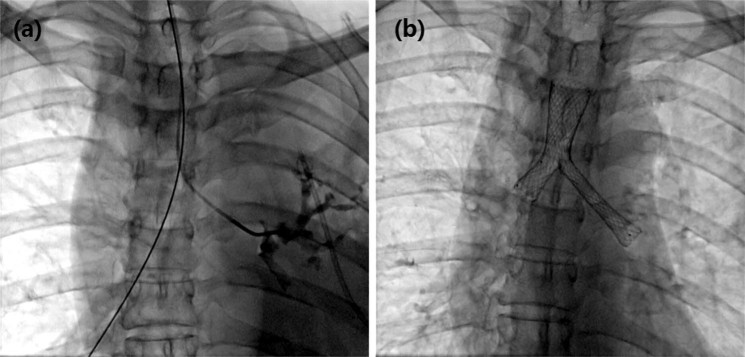
Operation of Y-type airway stents implantation. (**a**) Transcatheter airway angiography showed that left main bronchus was occluded and two stiff guide wires were introduced to both sides of bronchus. (**b**) The left lung permeability was increased under fluoroscopy immediately after placement of Y-shaped airway stent.

The procedures were performed under fluoroscopic guidance, without the use of bronchoscopy. Patients lay on the examination bed, with ECG monitoring. A gag was used to open the mouth. Oxygen was given via a nasal catheter and a sputum aspirator was prepared. After oral lidocaine surface anesthesia, a 0.035-inch hydrophilic guide wire (Cook Corporation, Bloomington, IN, USA) and a 5 F vertebral artery catheter (Cook Corporation) were introduced per os into the trachea or bronchus. Tracheography was performed to determine the location of bronchial stenosis. Exchange with stiff guide wire to one side bronchus, 9 F sheath tubes was placed along guide wire. Another 0.035 inch hydrophilic hard wire was introduced for Y-shaped or small Y-shaped stenting (Fig. 2). After the stent is successfully inserted, the sputum aspiration tube was introduced for sputum drainage to avoid suffocation. All patients were treated with atomization inhalation and anti-inflammatory drugs after stenting, and their vital signs were observed closely.

### Related treatments and follow-up

**Figure 3:**
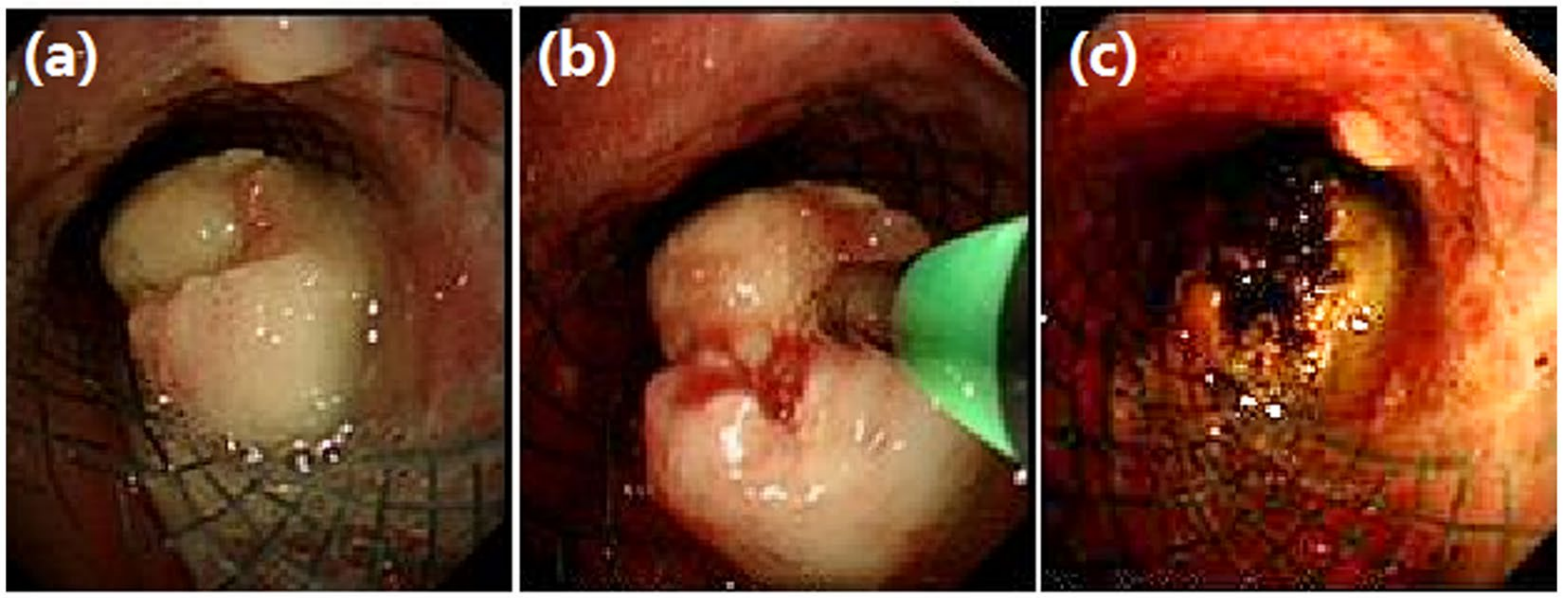
Bronchoscopic examination and treatment during follow-up. (**a**) Neoplasia could be seen in the airway stent. After endoscopic resection (**b**), the lumen was more patency than before (**c**).

After the improvement of pulmonary symptoms, 28 patients received chemotherapy. Of these, 14 patients underwent transarterial infusion chemotherapy. Nine patients received radiotherapy, including 6 patients who underwent ^131^I seed implantation. Five patients were placed in a nutrition tube to improve their nutritional status. After 2–5 days, the chest CT were re-examined to study the stent and atelectasis, and then re-examined regularly performed every 3 months. Patients with dyspnea were examined by bronchoscopy to observe the complications of stent. In cases of severe stenosis or obstruction, laser resection was performed after stent placement for post-stent overgrowth (Fig. 3).

### Statistical analysis

Data are presented as the mean ± SD or median with range. Student t test and survival curve were used. The *p*-value was considered statistically significant if *p* < 0.05.

## Results

### Technical and clinical results

**Figure 4:**
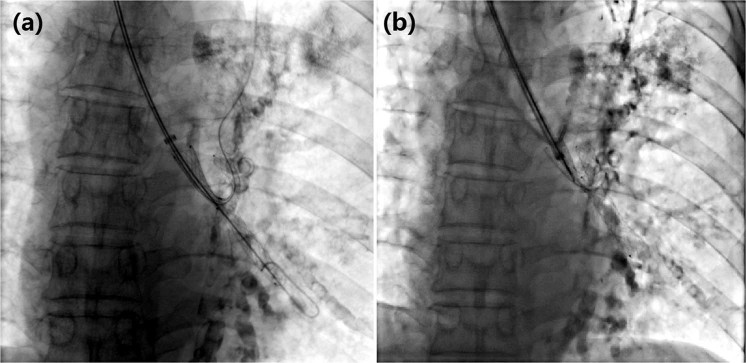
Placement and removal of a small y-shaped airway stent. (**a**) Due to poor expansion and deformation, a small Y-shaped airway stent placement failed. (**b**) The stent was immediately withdrawn by removal hook.

Thirty-eight stents were successfully implanted in 34 patients, included 29 Y-shaped stents, 4 small y-shaped stents, and 5 straight airway stents; only one small y-shaped stent failed in implantation due to insufficient dilatation and deformation, which was immediately remove by the hook^15^ (Fig. 4). The technical success rate of stenting is 97.1% (34/35). The lung of atelectasis was gradually restored and the oxygen saturation gradually increased after the stent placement; on the 2–5 days after stenting, 26 cases were confirmed full ventilation by chest CT, and 3 cases were partially ventilation, with a relieved dyspnea and improved respiratory function; the clinical success rate was 82.9% (29/35). Four of 6 cases without ventilation showed soft tissue obstruction in the stent branch and the remaining 2 cases showed a poor CT enhancement before stenting, although the stents were patency. The maximum enhancement CT value and the plain-enhanced CT difference were significantly higher in the patients with full ventilation, compared to patients without or partial ventilation (*p* < 0.01, Table 3). The patients with full ventilation showed an oxygen saturation of more than 94%, and the score of breath was significantly improved compared with those before stent, included 17 cases of grade 0, 11 cases of grade 1, and 5 cases of grade 2.

**Table 3:** CT enhancement and lung ventilation.

Viable	
Maximal CT value after enhancement	
Full ventilation (n = 26)	137.5 ± 12.5 (97–227)
None or partial ventilation (n = 9)	76.3 ± 8.5 (42–102)*
Plain-enhanced CT difference	
Full ventilation (n = 26)	97.8 ± 12.3 (50–179)
None or partial ventilation (n = 9)	39.7 ± 6.8 (18–62)*
Causes of none ventilation	
Soft tissue in stent	4 (66.7%)
Low enhancement	2 (33.3%)

*vs. Full ventilation *p* < 0.01.

### Complications and follow-up

**Figure 5:**
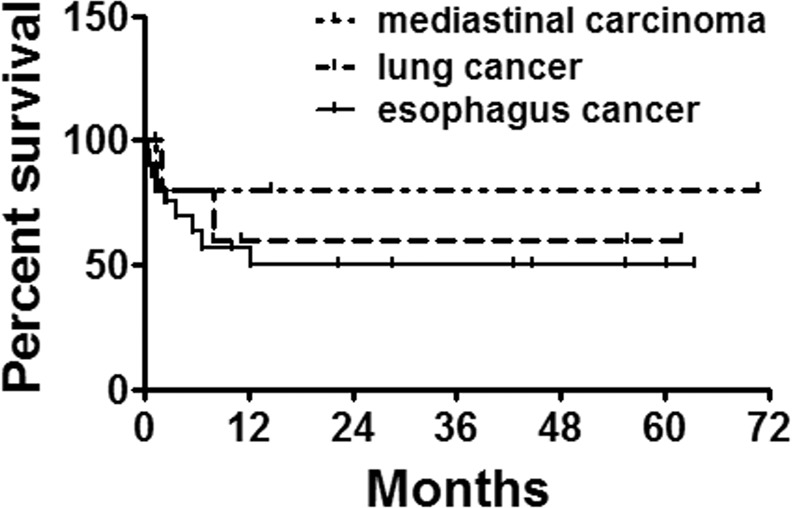
Survival follow-up. The survival rates of 3 months, 1 year and 2 years were 78.6%, 58.5% and 58.5%, respectively.

Perioperative non-operative related death was found in 2 patients, one died of asphyxia due to sputum obstruction and the other died of sudden cardiac accident. The average follow-up was 18.8 ± 4.0 months. Eight cases showed restenosis and received endoscopic laser resection, included 1 case underwent removal and 3 cases received second stenting. A large amount of sputum retention was observed in 10 patients, and received bronchofiberscope lavage and sputum aspiration. One patient showed strut fracture under fiberoptic bronchoscopy without any symptoms, and the patient refused further treatment. No stent migration was found. A total of 11 patients died after discharge, included 2 sudden cardiac death and 9 malignant tumors. The 3-month survival rates were 69.7%, 80%, 80% for esophagus cancer, lung cancer, and mediastinal carcinoma, respectively. The 5-year survival rates were 49.8%, 60%, 80% for esophagus cancer, lung cancer, and mediastinal carcinoma, respectively (Fig. 5).

## Discussion

Airway stenosis caused by primary pulmonary malignancies is associated with atelectasis^16^. Malignant obstructive atelectasis is caused by severe stenosis or occlusion of the trachea or bronchus and subsequently the disappearance of air in the alveoli of the lobes or lobules. The patients often presented progressive dyspnea, decreased oxygen saturation, and severe atelectasis. Atelectasis is a clinical emergency, which needs to be dealt with as soon as possible. Timely and effective treatment can make the atelectasis of the lung ventilated and expiratory dyspnea relieved, and win the opportunity for subsequent treatment^17^. Although SEMSs placement has become an effective method for airway stenosis^2–6^, few reports have described the treatment of atelectasis via SEMSs, except for some case reports. The bronchial stenting and high-frequency percussive ventilation was used for a case of atelectasis induced by extrinsic compression of aortic aneurysm^9^. Nakamura *et al*.^10^ described a case of left pneumothorax-induced atelectasis and underwent endobronchial stent placement. Serio *et al*.^11^ reported a case of compressed left main bronchus by huge heart dilation and left lung atelectasis was successfully treated by emergency bronchial stenting. However, stent treatment also brings potential fatal complications. Thus it is important to estimate the prior probability of success in the treatment of atelectasis via SEMSs placement.

Our study is one of the few study to report SEMSs for the treatment of obstructive atelectasis. In the present study, 29 Y-shaped stents, 4 small y-shaped stents, and 5 straight airway stents, were selected and successfully implanted according to the location of airway lesions. Only one small y-shaped stent failed in implantation due to insufficient dilatation and deformation, for a technical success rate of 97.1%. For the choice of stent type, the tubular stent may be easily migrated, while the Y-type airway stent straddling the airway bifurcation is not easy to migration. There is no stent displacement in the present study. All cases showed airway stenosis with no fistula, so bare stent rather than covered stent was selected in this study. However, the bare stents may increase the risk of post-stent overgrowth. Covered stents have advantage in preventing post-stent ingrowths, while having disadvantage in stent migration. Twenty-six cases of full ventilation were confirmed by CT 2 to 5 days after stenting, 3 cases showed partial ventilation and the clinical success rate was 82.9%. Soft tissue obstruction in stent branches was found in 4 out of 6 patients without ventilation. We speculated that a large number of mucous sputum embolus and necrotic tumor tissue were the main causes of failure.

Obvious enhancement of atelectasis tissue on CT images before stenting indicates that there is still blood supply in the lungs without losing the function of oxygen intake, it is expected that the lungs will be successfully ventilated after stent placement. In the present study, we found that the maximum enhancement CT value and the plain-enhanced CT difference were significantly higher in the patients with full ventilation, compared to patients without or partial ventilation. The poor enhancement of CT before stenting is quite poor in the 2 cases of the patients without ventilation. The results suggest that CT enhancement can be used to evaluate the enhancement of atelectasis lung tissue, which is helpful to estimate whether the atelectasis can be successfully ventilated after stent implantation.

SEMSs placement can effectively relieve symptoms, improve pulmonary ventilation and stabilize cardiovascular conditions for further treatments^11,17–19^. However, stent treatment also brings potential fatal complications^12–14^. Hachiya *et al*. even reported the potential risk of creating obstructive atelectasis in two cases after stenting^2^. The most common complications of stent implantation are sputum retention and stent restenosis caused by tissue proliferation. Other complications included stent migration, intraluminal tumor growth, tracheal fistula, stent fracture, and so on^18^. In addition, it has also been reported that the incidence of stent-associated respiratory tract infection after stent placement is 20%^20^. In the present study, no stent rupture, tracheal fistula and stent associated respiratory tract infection were found. Severe restenosis in stents was found in 8 patients during follow up, and required fiberoptic bronchoscopy or re-stent therapy. Difficulty in sputum drainage was found in 10 patients, airway obstruction was relieved and symptoms disappeared after aspiration of sputum by fiberoptic bronchoscopy.

After the improvement of pulmonary symptoms, 28 patients received chemotherapy. Of these, 14 patients with tumor underwent transarterial infusion chemotherapy. Nine patients received radiotherapy, including 6 patients who underwent ^131^I seed implantation. Five patients showed atelectasis after surgical resection for esophageal cancer and lung cancer. All above factors might result in good outcomes of survival rates.

There are several weaknesses in our study. The endoluminal obstruction should be treated by debulking, laser, or electrocautery sometimes followed by stents, while the extraluminal obstruction could be solely treated by stents. We aimed to use the method of stent placement to ventilate atelectasis as soon as possible. All the patients were atelectasis caused by malignant tumor, because of the tumor extensive invasion, it is difficult for us to distinguish whether it is simple extraluminal obstruction or endoluminal stenosis. Although most patients receive transarterial chemotherapy and particle seeds implantation after stenting, the bare airway stent itself can only relieve the patient’s symptoms. Particle-carrying SEMSs may be a better choice. Stent removal was needed in some patients due to complications such as stent restenosis, which may cause tracheobronchial injury^15,21^, and the biodegradable stent can be avoided the removal injury^22^.

In conclusion, although SEMSs have their limitations, they play an indispensable role in the treatment of obstructive atelectasis, and may be a promising strategy for reopening atelectatic lung tissue. The preoperative CT enhancement played an important role in estimating the prior probability of success in the treatment of atelectasis via SEMSs placement

## Data Availability

No datasets were generated or analyzed during the current study.
